# A Case of Congenital Duodenal Aresia

**Published:** 1911-12

**Authors:** Francis Roe, Ernest H. Shaw

**Affiliations:** La Clinique Infantile; La Clinique Infantile


### A Case of Congenital Duodenal Aresia

By FRANCIS ROE and ERNEST H. SHAW
in *La Clinique Infantile*

A DOCTOR was called to see a woman aged 36, upon her third pregnancy. On his arrival the woman had borne a male child, well constituted. Labor had been easy, and there was need of a nurse only an hour before delivery.

Mother or nurse did not complain of anything abnormal concerning the baby, except that it did not appear to take the breast with much appetite. Four days later, the baby cast up its milk and brown substances. The child appeared well and no alarm was felt. The next day the child died suddenly. All the preceding night and day the child had vomited considerable quantities of brown substances and had a number of stools during the evening.

The doctor was unable to certify the cause of death, and obtained permission to examine the body. There were no external signs of disease, except a distention and hardness of the abdomen. The lungs and heart were normal. The distended stomach occupied the whole abdominal cavity; towards the pyloric extremity there appeared a constriction with a protuberance containing a brownish liquid substance. The intestines were not distended. The other organs were normal.

Upon the examination of a piece comprising the stomach and duodenum stuffed with cotton and fixed in the normal position he found: A large stomach with a a very serrated constriction at the junction of the superior 2-3 and the distal 1-3; the opening of the oesophagus in its normal position and at the other extremity a narrow tube issuing from the middle of an extremity in the caecum; the narrowed portion of the stomach was the pylorus, for a well marked muscular ring about 1-3 inch large represented the pyloric sphincter. The dilated portion on the other side was the duodenum, which gradually dilated till it formed a tube 1½ inch in diameter, then terminated abruptly, where the lumen of the duodenum was completely obstructed. From the exterior of the caecum, the part was a narrow tube with a thin wall, about ¼ inch in diameter. The mucous membrane was irregularly wrinkled . There was a slight external constriction at the union of the two parts. The appearance presented by this narrow tube issuing from the dilated portion of the duodenum resembled the appendix issuing from the caecum. Only towards the pylorus was the duodenum covered by peritoneum. A narrow duct stained with bile penetrated the posterior part of the duodenal wall just at the beginning of the narnow part; a hair could be introduced into the tube. But it could be made to penetrate into the lumen of the intestine. This was the common bile duct and must have existed as the passage during foetal life since there was meconium. But it was difficult to demonstrate because of hardening. Another tube on the side of the preceeding served to represent the pancreatic duct. A hair could be introduced into the tube but did not pass into the duodenum. On the interior of the pylorus was found a depression, narrow and deep, about 5-16 inch long by 2-16 inch deep, perpendicular to the large axis of the pylorus and separated from the origin of the duodenum, by a bridge of mucous membrane 1/8 inch large. A piece of it was cut and the mucous membrane was seen to dip in deeply; the appearance of the section leading into the depression recalls the section of a laryngeal ventricle. The pyloric sphincter about 1/8 inch thick terminated brusquely at the duodenal junction, descended gradually towards the cardia. The stomach was very dilated and its walls hypertrophied. The lesser curvature was 2½ inches long, from the oesophagus to the pylorus. The greater curvature was 7 inches. The greatest width from the lesser to the greater curvatures was 1¾ inches. The duodenum was 2 inches long, from the superior extremity of the duodenum to the constriction, 3½ inches to the inferior extremity.

MICROSCOPIC EXAMINATION.

At the bottom of the depression of the pylorus is an opening of a large branched duct, covered by glandular cells. Around this duct are large bands of gland tissue, irregularly distributed. The glandular mass seems to have penetrated the depth of the muscular wall. The specimen is interesting and rare. Professor A. Keith, who has sought for literature on this malformation has found seven other observations. He cites the opinion of Bland Sutton that Atresia is probably consistent with the development of embryonal cells of the liver and pancreas and that it is produced at the junction of the two morphologic parts of the alimentary canal: the anterior and lesser grooves. Forster says that the duodenal segment of Vater is closed by the proliferation of its epithelial lining, during the greatest part of the second month. If the epithelial obstruction is not destroyed in proportion to development, an occlusion at this point is produced. The specimen has been deposited at the museum of the Royal College of English Surgeons.

**From La Clinique Infantile (1910)**

The thermic regulations in the healthy nursling and in the babe afflicted with digestive troubles.

H. Heim and M. K. John (of Budapest) state that a little pallor rise in the childs temperature is indicative of digestive disturbances; the thermic oscillations are usually accompanied by irregularities in the curve of its feet.

The healthy child reacts against external cold by a peripheral vaso-constriction; if it is left for five minutes in a room whose temperature is from 21° to 23°C, the extremities become colder, while the internal temperature, taken in the rectum, rises from 1-12° to 2-12°.

If the same is done with a child affected by decomposition, the temperature at the end of five minutes will fall about 7-10° and five minutes later about 1.2°.

**From La Clinique Infantile**

Researches on the influence of turnips on the quality of milk.

1. The disagreeable taste and odor of the milk of cows nourished on turnips has been found to be caused by digestive disturbances and by the difficulty which results from keeping the cows absolutely clean, and the atmosphere of the stable pure and fresh.

2. It has also been found that the acidity of the milk becomes augmented, due to the increased quantity of phosphoric acid that has passed into the milk.

3. The increased acidity of the milk gives it a greater capacity to coagulate. It is not impossible that the increased coagulability may be due to the greater amount of calcium that has passed into it.

